# Large Vallecula Epiglottica Lipoma: A Rare but Fatal Cause of Dysphagia

**DOI:** 10.7759/cureus.30250

**Published:** 2022-10-13

**Authors:** Siti Nurafiqah Sharudin, Thilagam Thangavelu, Siti Nor'Ain Roslim, Shahrul Hitam, Marina Mat Baki

**Affiliations:** 1 Otorhinolaryngology-Head and Neck Surgery, Universiti Kebangsaan Malaysia Medical Centre, Kuala Lumpur, MYS; 2 Otorhinolaryngology-Head and Neck Surgery, Hospital Ampang, Selangor, MYS

**Keywords:** epiglottis, excision, fat, dysphagia, voice, vallecula, lipoma

## Abstract

Lipoma is a benign mesenchymal tumor with 13% occurrence in the head and neck region. Despite being the commonest type of tumor, it only accounts for 0.6% of the upper aerodigestive tract. We report a case of a 41-year-old gentleman who presented with progressively worsening dysphagia and a muffled voice. A flexible nasopharyngeal endoscope showed a solitary well-lobulated pedunculated cystic-looking mass occupying the oropharynx arising from the vallecular space and lingual surface of the epiglottis with a partially seen mobile posterior vocal cord. CT of the neck showed a single lesion with fat attenuation in the right vallecula and right lateral lingual epiglottis. The patient underwent endoscopic surgical excision and fully recovered post-operatively. Although rare, lipoma must be considered one of the differential diagnoses of midline laryngeal mass. Therefore, prompt excision needs to be performed to prevent a catastrophic outcome.

## Introduction

Lipoma is a common benign mesenchymal tumor, with an occurrence of 13% in the head and neck region. However, its incidence is rare in the upper aerodigestive tract accounting for only 0.6% of all benign neoplasms [1]. The slow-growing characteristic of the tumor leads to a delay in detection. However, it can be fatal depending on the size and location of the tumor [2-3]. In this article, we report our successful management of a case of vallecula lipoma in a middle-aged gentleman.

## Case presentation

A 41-year-old non-smoker gentleman with underlying diabetes mellitus presented with a complaint of dysphagia and muffled voice for two years, gradually worsening over one month and associated with foreign body sensation in the throat and loud snoring. His appetite was good, but he could only tolerate a small amount of solid food with each meal. He denied neither any history of shortness of breath, loss of weight, recent infection, nor a history of foreign body ingestion prior to it.

**Figure 1:**
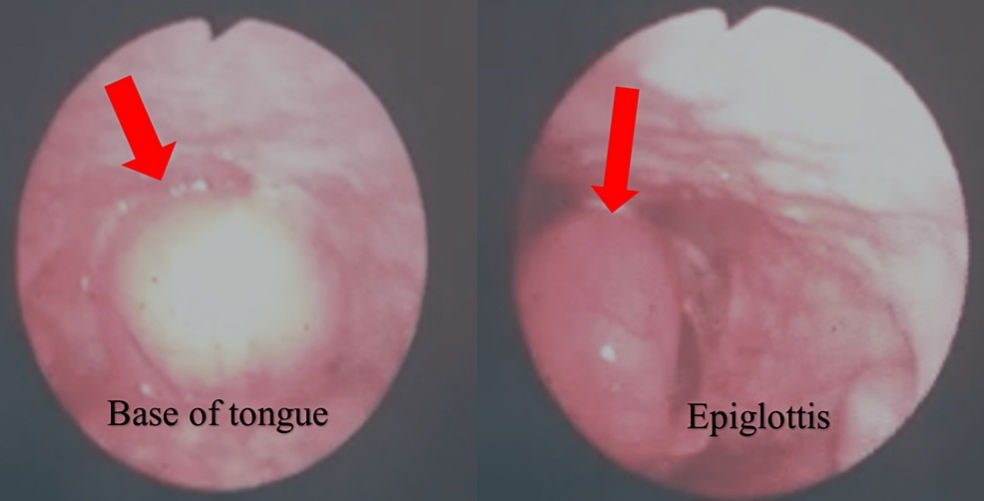
FNPLS showed solitary, well-lobulated and pedunculated cystic-looking mass occupying the oropharynx (arrow). FNPLS: Flexible naso-pharyngo-laryngoscopy.

On examination, he was stertorous. Oropharyngeal examination revealed a single, well-lobulated, cystic-looking mass that plummeted back and forth within the oropharynx. Its pedunculated nature was apparent. Other ears, nose, neck, and systemic examinations were unremarkable. A flexible naso-pharyngo-laryngoscopy (FNPLS) showed a solitary well-lobulated and pedunculated cystic-looking mass, which appeared benign, occupying the oropharynx arising from vallecula space and lingual surface of epiglottis (Figure 1).

**Figure 2:**
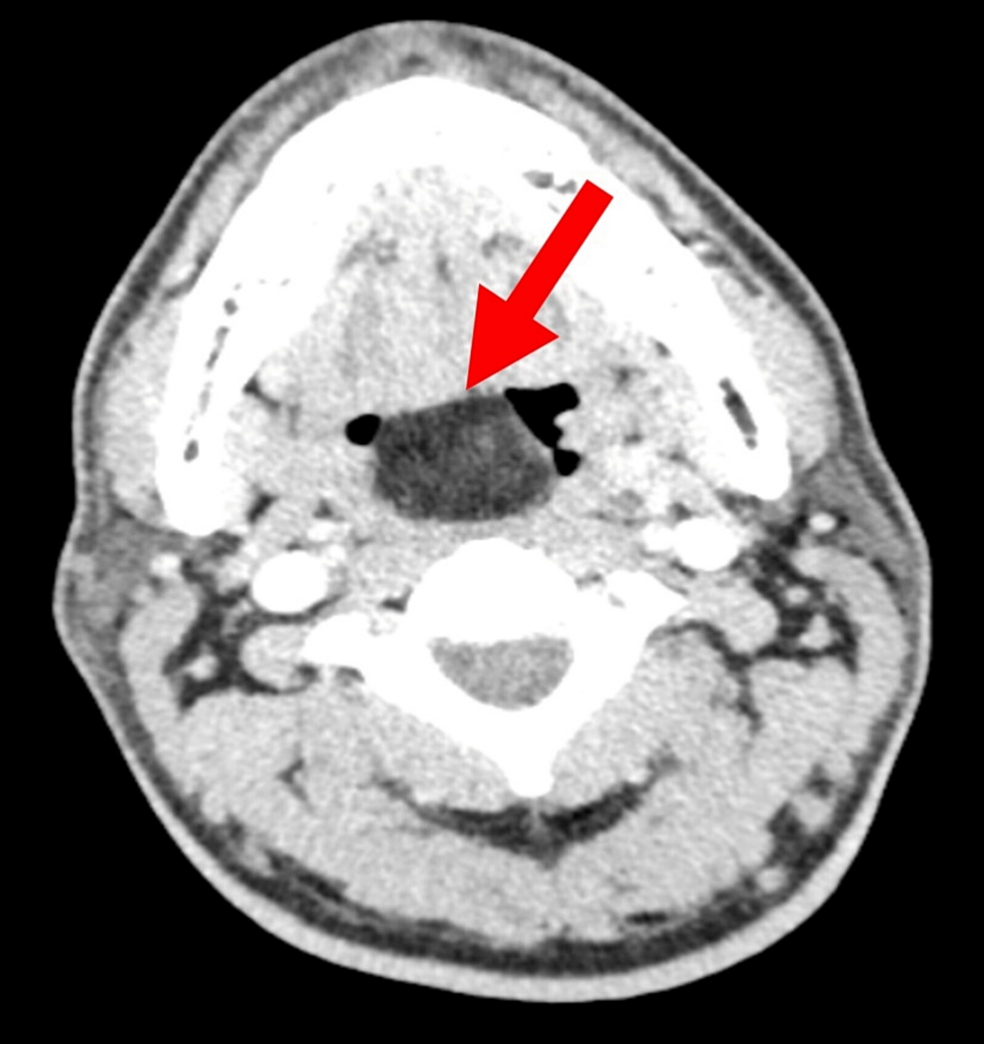
Axial CECT neck showed that the lesion measured approximately 2.6 x 2.6 x 3.0 cm. It had a mean attenuation of -72 to -61HU (suggestive of fat component). CECT: Contrast-enhanced computed tomography.

**Figure 3:**
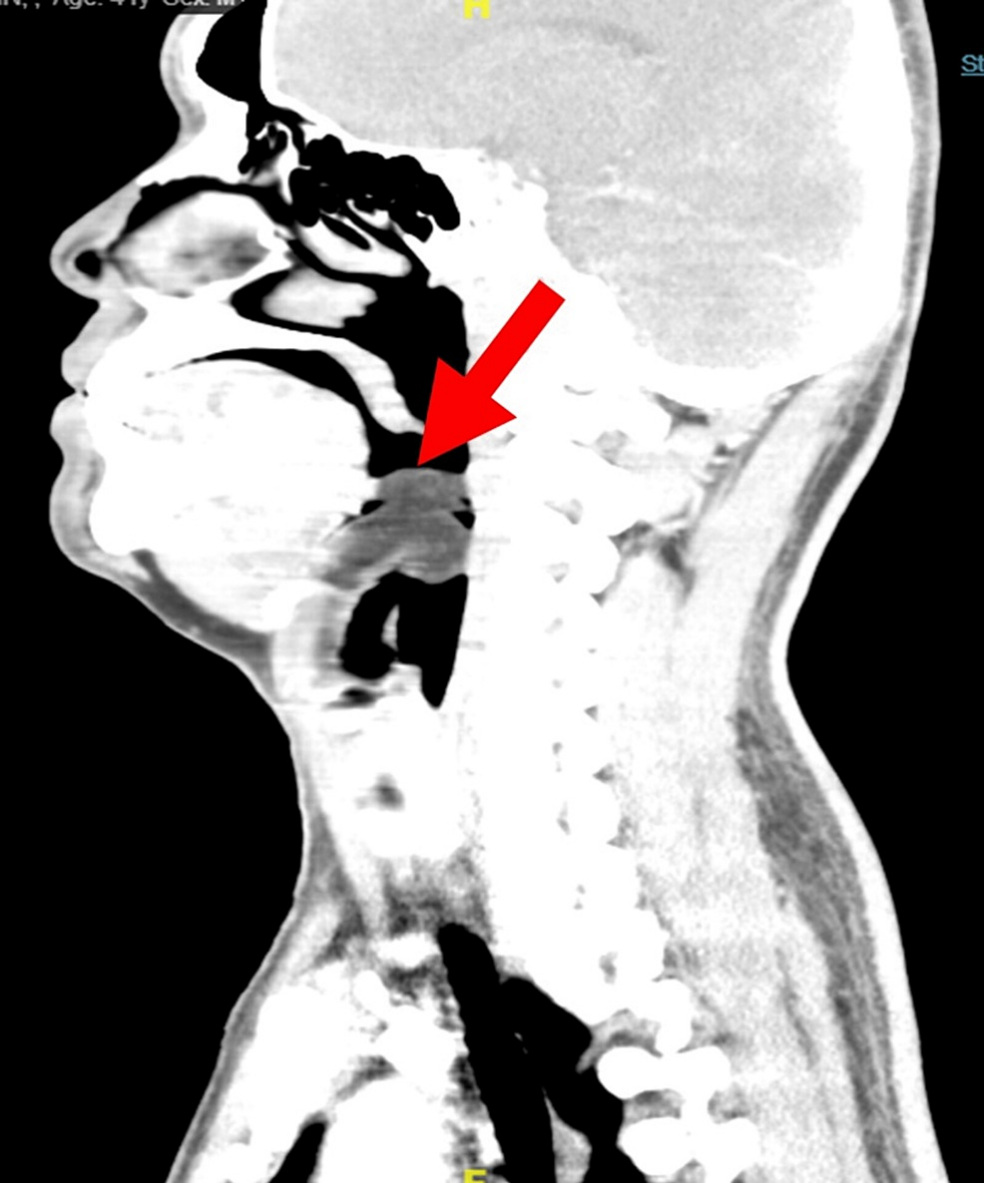
The same lesion as it appears on sagittal reformatted image. It is broad based attached to right lateral side of the lingual surface of epiglottis.

**Figure 4:**
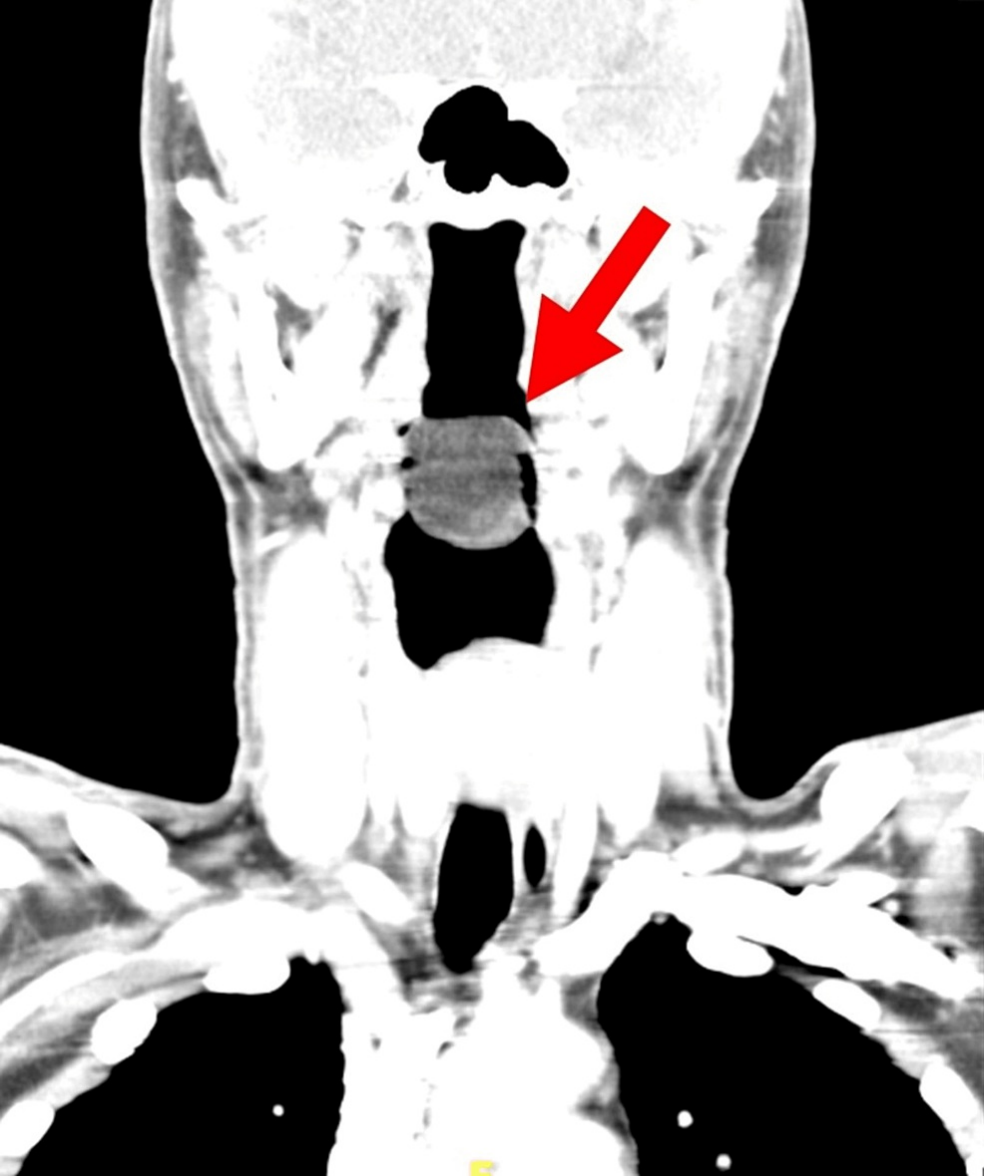
The same lesion as it appears on coronal reformatted image showing narrowing of the oropharynx. The hypopharynx is spacious.

Both posterior vocal folds were partially seen and were mobile. A contrast-enhanced computed tomography (CECT) of the neck showed a well-defined pedunculated smooth margin homogenous hypodense lesion arising from the right vallecula space and right lateral side of the lingual epiglottic surface and extending superoposteriorly into the oropharynx causing narrowing of the oropharynx. A clear fat plane was seen on the posterior pharyngeal wall, the base of the tongue, the uvula, and both lingual tonsils. The lesion measured approximately 2.6 x 2.6 x 3.0 cm. It had a mean attenuation of -72 to -61HU (suggestive of fat component) with no significant enhancement. Neither solid, cystic component nor calcification was seen (Figures 2-4).

The patient was advised of the risks of open tracheostomy before the surgery. The patient gave consent for the procedure. Examination under anesthesia, direct laryngoscopy, and endoscopic excision of the mass was done. Before induction, the Ear Nose Throat (ENT) team prepared for an open tracheostomy under local anesthesia if an intubation attempt failed. During induction, the anesthetists' team had difficulty securing the airway due to the prolapsed mass and its slippery property, causing the desaturation of the peripheral oxygen to almost 60%.

**Figure 5:**
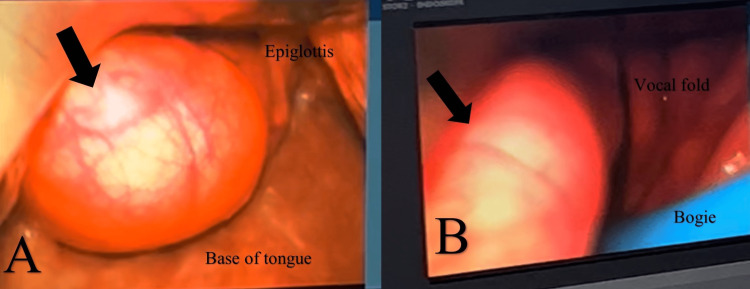
For better visualization, a C-MAC video laryngoscope was used, and the mass was pushed to the lateral side. Intubation was done using an endotracheal tube size of 7.5 cm with the help of a bogie (arrow).

After failed awake fibreoptic nasal intubation, the ENT team proceeded with intraoral endotracheal intubation using a direct visualization with a C-MAC video laryngoscope. With better visualization using the C-MAC, the mass was pushed to the lateral side, and intubation using an endotracheal tube size of 7.5 cm with the help of a bogie was successful without any desaturation (Figure 5A-5B).

**Figure 6:**
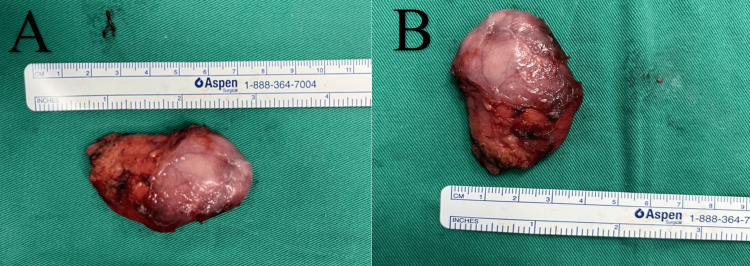
Single solid mass measuring 6x4 cm removed intraoperatively.

Intraoperatively revealed a single solid mass measuring 6x4 cm with a broad-based peduncle arising from the right vallecula and right lateral lingual surface of epiglottis (Figure 6A-6B).

**Figure 7:**
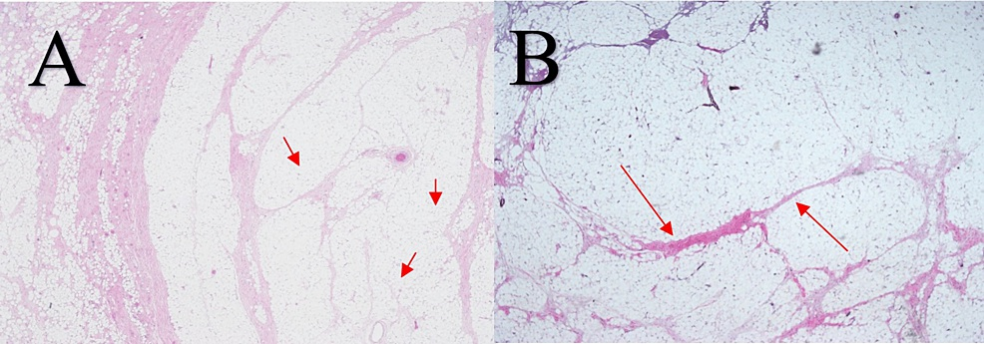
Lobules of mature univacoulated adipocyte intersected in area by fibrous septa (arrow) (H&E x40).

The mass was removed using monopolar diathermy. Histologically yielded lobules of mature univacoulated adipocytes intersected in the area by fibrous septa with no lipoblast or atypical stromal cell present. It is covered by non-keratinizing stratified squamous epithelium with the presence of a seromucinous gland and cartilage in the subepithelial region (Figure 7A-7B).

The procedure was uneventful. He was discharged the next day, made a full recovery within two weeks, and was able to return to work.

## Discussion

Vallecula is a potential space located anterior to the epiglottis, forming the floor of the oropharynx. It is separated from the base of the tongue via the median glossoepiglottic fold and bound laterally via the lateral glossoepiglottic fold [4-5]. Histologically, vallecula shows abundant lymphoid and glandular tissue; thus, blockage of the submucosa duct leads to a vallecula cyst, the most typical mass lesion is seen (10-20%) [6-7]. The occurrence of lipoma at the upper aerodigestive tract is rare as it accounts for only 0.6% of all benign neoplasms. Mostly this condition is seen on the cheek region, tongue, floor of the mouth, buccal sulcus, palate, lip, and gingiva [8]. Murty KD et al. postulated that the etiology of lipoma presented in this region is due to fibroblast being a multipotential cell whereby it may differentiate into a fat cell and form true lipoma [9].

Benign lipoma is a slow-growing tumor in nature [2]. Clinically, patients presented with features related to oropharyngeal and hypopharyngeal obstruction, such as snoring, change of voice, dysphagia, and foreign body sensation [10]. Alas, if the mass is large enough, it can lead to upper airway obstruction and stridor; thus, it should not be taken lightly and must be addressed immediately [10]. Our patient had dysphagia and a muffled voice for two years before his presentation, suggesting that the lesion was slow-growing. However, as the mass increased until it occupied 70-80% of the laryngeal space, the patient started developing dysphagia and foreign body sensations, forcing him to seek medical attention.

Due to its location, a laryngeal mass may pose a diagnostic and surgical dilemma; thus, imaging of the lesion is essential. A retrospective study done by Wang GX et al. in the pediatric population concluded that FNPLS combined with USG of the neck is sufficient to diagnose and exclude ectopic thyroid gland as it is commonly congenital and benign [11]. However, differential diagnoses of midline laryngeal mass in adult populations can be benign or malignant, such as lingual thyroid cysts, thyroglossal cysts, mucous retention cysts, dermoid cysts, salivary gland neoplasm, lymphangioma, hemangioma, benign lipoma, and liposarcoma [12]. Furthermore, with the advancement of modern imaging such as CECT, diagnosis of lipoma is adequate owing to its typical characteristic such as homogenous non-enhancing low attenuation measure between -65 to -125 HU with clear fat plane from adjacent structure [12-13]. This feature was seen in our patients as well. On the other hand, MRI is more sensitive and accurate in evaluating soft tissue lesions [14], together with appraising the submucosa region and perineural invasion if a more sinister disease is suspected [12]. However, due to its benign appearance and characteristic seen in CECT, our patient did not proceed with MRI.

Histology remains the gold standard in diagnosing lipoma. It is commonly composed of mature univacoulated adipocytes intersected in areas by fibrous connective tissue septa. It may be categorized into classic lipoma or other variants such as fibro lipoma, angiolipoma, spindle cell lipoma, pleomorphic, myxoid, sialolipoma, and intramuscular lipomas [13]. Although classic lipoma is the most typical type of lipoma, the rarity of its location in the vallecular space makes it a unique differential in our case.

A large vallecula lipoma may possess difficult intubation due to its location and slippery nature. Good communication between the ENT and anesthetists team is vital to overcome the expected difficult intubation. Alternative approaches for intubation and a standby emergency tracheostomy procedure need to be clearly emphasized. Therefore, an experienced anesthesiologist and otorhinolaryngologist are needed to facilitate and ease the procedures to reduce fatal complications such as total upper airway obstruction by a large vallecula mass. Besides endotracheal intubation using a direct visualization with a C-MAC video laryngoscope, as in our patient, other techniques using awake flexible fiberoptic intubation [14-15] also have been proposed.

Complete surgical excision is the mainstay treatment, as recurrence may develop if incomplete excision is done [9]. A transoral approach is preferable to the open approach as lipoma is usually well encapsulated, and complete excision can be attained [1]. Furthermore, rapid recovery and laryngeal function can be restored with fewer late complications [1]. Even though lipoma is a benign disease, due to its location, precautions must be taken during anesthesia induction, whereby the airway is at risk. In such an event, an emergency tracheostomy must be done to secure the airway [3].

## Conclusions

Vallecula lipoma is a rare, slow-growing tumor and can carry a high risk of upper airway obstruction due to its location. Although rare, lipoma must be considered one of the differential diagnoses of midline laryngeal mass. CT and MRI are the primary imaging modalities to characterize these lesions, establish the origin of the tumor, and delineate the extent of the disease. In addition, complete excision of the lesion is essential to prevent a recurrence.
